# High-energy interference-free K-lines synchrotron X-ray fluorescence microscopy of rare earth elements in hyperaccumulator plants

**DOI:** 10.1093/mtomcs/mfad050

**Published:** 2023-08-17

**Authors:** Antony van der Ent, Dennis Brueckner, Kathryn M Spiers, Ken Vidar Falch, Gerald Falkenberg, Clément Layet, Wen-Shen Liu, Hong-Xiang Zheng, Marie Le Jean, Damien Blaudez

**Affiliations:** Université de Lorraine, INRAE, LSE, F-54000 Nancy, France; Laboratory of Genetics, Wageningen University and Research, The Netherlands; Centre for Mined Land Rehabilitation, Sustainable Minerals Institute, The University of Queensland, Australia; Deutsches Elektronen-Synchrotron DESY, Hamburg, Germany; Deutsches Elektronen-Synchrotron DESY, Hamburg, Germany; Deutsches Elektronen-Synchrotron DESY, Hamburg, Germany; Deutsches Elektronen-Synchrotron DESY, Hamburg, Germany; Université de Lorraine, INRAE, LSE, F-54000 Nancy, France; Université de Lorraine, CNRS, LIEC, F-54000, Nancy, France; School of Environmental Science and Engineering, Guangdong Provincial Key Laboratory of Environmental Pollution Control and Remediation Technology, Sun Yat-sen University, China; School of Environmental Science and Engineering, Guangdong Provincial Key Laboratory of Environmental Pollution Control and Remediation Technology, Sun Yat-sen University, China; LIEC, Université de Lorraine, CNRS, Metz, France; Université de Lorraine, CNRS, LIEC, F-54000, Nancy, France

**Keywords:** high-energy detector, hyperaccumulator, lanthanides, L-lines, REEs

## Abstract

Synchrotron-based micro-X-ray fluorescence analysis (µXRF) is a nondestructive and highly sensitive technique. However, element mapping of rare earth elements (REEs) under standard conditions requires care, since energy-dispersive detectors are not able to differentiate accurately between REEs L-shell X-ray emission lines overlapping with K-shell X-ray emission lines of common transition elements of high concentrations. We aim to test REE element mapping with high-energy interference-free excitation of the REE K-lines on hyperaccumulator plant tissues and compare with measurements with REE L-shell excitation at the microprobe experiment of beamline P06 (PETRA III, DESY). A combination of compound refractive lens optics (CRLs) was used to obtain a micrometer-sized focused incident beam with an energy of 44 keV and an extra-thick silicon drift detector optimized for high-energy X-ray detection to detect the K-lines of yttrium (Y), lanthanum (La), cerium (Ce), praseodymium (Pr), and neodymium (Nd) without any interferences due to line overlaps. High-energy excitation from La to Nd in the hyperaccumulator organs was successful but compared to L-line excitation less efficient and therefore slow (∼10-fold slower than similar maps at lower incident energy) due to lower flux and detection efficiency. However, REE K-lines do not suffer significantly from self-absorption, which makes XRF tomography of millimeter-sized frozen-hydrated plant samples possible. The K-line excitation of REEs at the P06 CRL setup has scope for application in samples that are particularly prone to REE interfering elements, such as soil samples with high concomitant Ti, Cr, Fe, Mn, and Ni concentrations.

## Graphical Abstract

**Graphical Abstract:**
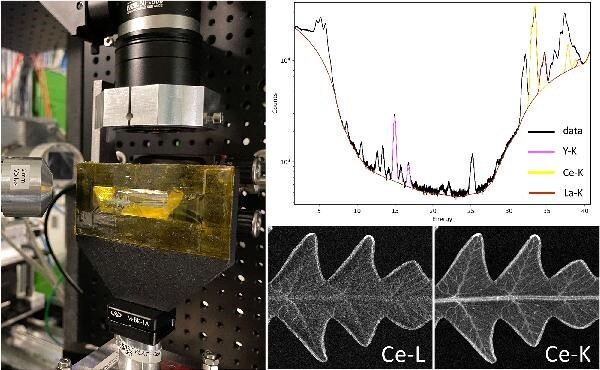
Analytical setup of the experiment at beamline P06 (DESY) with high-energy XRF spectrum and Ce L-line and K-line maps.

## Introduction

The group of rare earth elements (REEs) consists of 17 metallic elements including the 15 lanthanides, yttrium (Y), and scandium (Sc). Even though the average REE concentration of global soils is ∼150 mg kg^−1^ dry weight (DW), most plants usually have very low prevailing concentrations of REEs in their shoots (<5 mg kg^−1^ DW).^1,2^ Hyperaccumulator plants can accumulate and tolerate high concentrations of toxic elements in their living shoots. Thus far, ∼700 plant species have been reported globally to hyperaccumulate a large variety of metals and metalloids, less than 25 plant species are known as REE hyperaccumulators.^3,4^ REE hyperaccumulation is defined as plants that have in excess of 1000 mg kg^−1^ DW total REEs (tREEs) in their shoots.^5,6^ REE hyperaccumulation appears to be an erratic phenomenon and is likely a consequence of inadvertent uptake using existing mechanisms for uptake of K, Na, Ca, Mn, and Al.^7,8^ For example, the addition of Ca, Mn, and Al can significantly inhibit the uptake and accumulation of REEs in a hydroponically grown hyperaccumulator *Phytolacca americana*.^9^ Similar to the detoxification of nickel, zinc, cadmium, etc., the sequestration of REEs in hyperaccumulator plant leaves was suspected to depend on organic acids complexation and cell walls fixation.^10,11,12^ However, studies also revealed the possible role of silicon in dealing with the high concentration of REEs in the best-known hyperaccumulator *Dicranopteris linearis*.^13,14^ Nevertheless, the uptake and transport mechanisms of REEs are yet to be fully understood, which limits the development of phytotechnologies by using these REE hyperaccumulator plants.

Except for cerium (Ce) (+3 and +4 valences) and europium (Eu) (+2 and +3 valences), the REEs are a group of trivalent elements with similar physio-chemical properties.^15^ However, their minor differences in ionic radii with increasing atomic number result in the fractionation of REEs across various biogeochemical processes.^1^ The lanthanides are generally classified into light REEs (LREEs: La–Eu) and heavy REEs (HREEs: Gd–Lu). In soil-plant systems, it was reported that the HREEs enrichment in the leaves of wheat is ascribed to preferential scavenging of REEs by phosphate deposition and cell wall fixation in the roots^16^; while the preferential transport of HREEs in the xylem sap plays a role in HREE enrichment in *P. americana* leaves.^9^ These studies imply that the plants seem to have evolved the ability to differentiate various REEs and information on differential behavior of REEs in biological tissues is of significance to gain a deeper understanding of the ecophysiological and biological behaviors of REEs in plant systems.

The distribution of REEs is most frequently analysed with laser ablation–inductively coupled plasma–mass spectrometry (LA–ICP–MS) but the destructive nature of this technique is a major disadvantage. In contrast, synchrotron-based micro-X-ray fluorescence analysis (µXRF) is a nondestructive and highly sensitive technique that has been used to investigate numerous plants.^17^ Previously, we showed that it was possible to differentiate between REEs (La, Ce, Sm, Gd, Yb) in fronds of *Dryopteris erythrosora*, harboring a moderate/high accumulation of REEs (350 to 2000 mg kg^−1^ DW) but with relatively high concentrations of interfering elements (Fe, Mn).^18^ Conversely, differentiation between La and Ce distribution was not possible in *D. linearis* samples with high prevailing concentrations of total REEs (tREEs) and interfering elements (Fe, Mn).^19^ This is a consequence of the reliance on detectors [silicon drift detectors (SDDs) with an energy resolution 125 eV at 5.9 keV, increasing with count rate] that are typically not able to accurately determine the line intensities of the various REEs (due to their overlapping L-shell X-ray emission lines that are separated only by tens of eVs) in the presence of the K-lines of transition elements (such as Ti, Fe, Mn, Cr ).^20^ This interference of K-lines of transition elements with L-lines of REEs can be avoided by excitation of the interference free K-lines of the REEs (38.92 keV K absorption edge for La). However, most synchrotron µXRF beamlines are limited to below 20 keV incident energy and high photon energies are the domain of high electron energy storage rings like ESRF, APS, Spring 8, and PETRA III.

The Hard X-ray Micro/Nanoprobe beamline P06 at PETRA III has recently been upgraded for microscopic XRF scanning microscopy with sub-micrometer resolution up to 35 keV incident beam energy, and in this study we extend the capabilities of P06 experiment to even higher energies. We use a combination of compound refractive lens (CRL) optics to obtain a focused incident beam with an energy of 44 keV and use an extra-thick SDD optimized for high-energy X-ray detection. The use of an incident beam of 44 keV should enable to excite the K-lines of yttrium (Y), lanthanum (La), cerium (Ce), praseodymium (Pr), and neodymium (Nd) without any interferences due to line overlaps. In this study we test a hyperaccumulator (*D. linearis*) of very high tREE status (>2000 mg kg^−1^ DW) as a target for the high-energy REE excitation and compare with low-energy REE excitation of the exact same sample. The high-energy X-ray optics performance is described in general with respect to future high-energy microfocus experiments which deserve higher spatial resolution than the current medium resolution application.

## Materials and methods

### Plant material and specimen preparation for ICP–OES and ICP–MS elemental analysis

The *D. linearis* samples were separated into roots and shoots after washing with deionized water and then desorbed in 5 mM CaCl_2_ solution for 15 min. The samples were then dried at 120°C for 2 h and then at 60°C for 3 d. The rhizosphere soil sample of *D. linearis* was collected and air-dried for 2 weeks. Then, the samples were ground to pass through a 0.15 mm screen prior to acid digestion. The plant samples were digested with 6 mL 50% HNO_3_ and 2 mL H_2_O_2_ at 105°C following USEPA Method 3050B. For soil sample, 8 mL aqua regia was added and the mixtures were digested in a microwave (MARS6, CEM, USA). All digestates were filtered through 0.45 µm membranes. ICP–optical emission spectrometry (ICP–OES, Thermo, USA) was used for K, Ca, Na, Mg, Al, Mn, and Fe analyses, while ICP–MS (NexION 300D, PerkinElmer, USA) was used for the REEs.

### Plant material and specimen preparation for synchrotron µXRF analysis

**Fig. 1.**
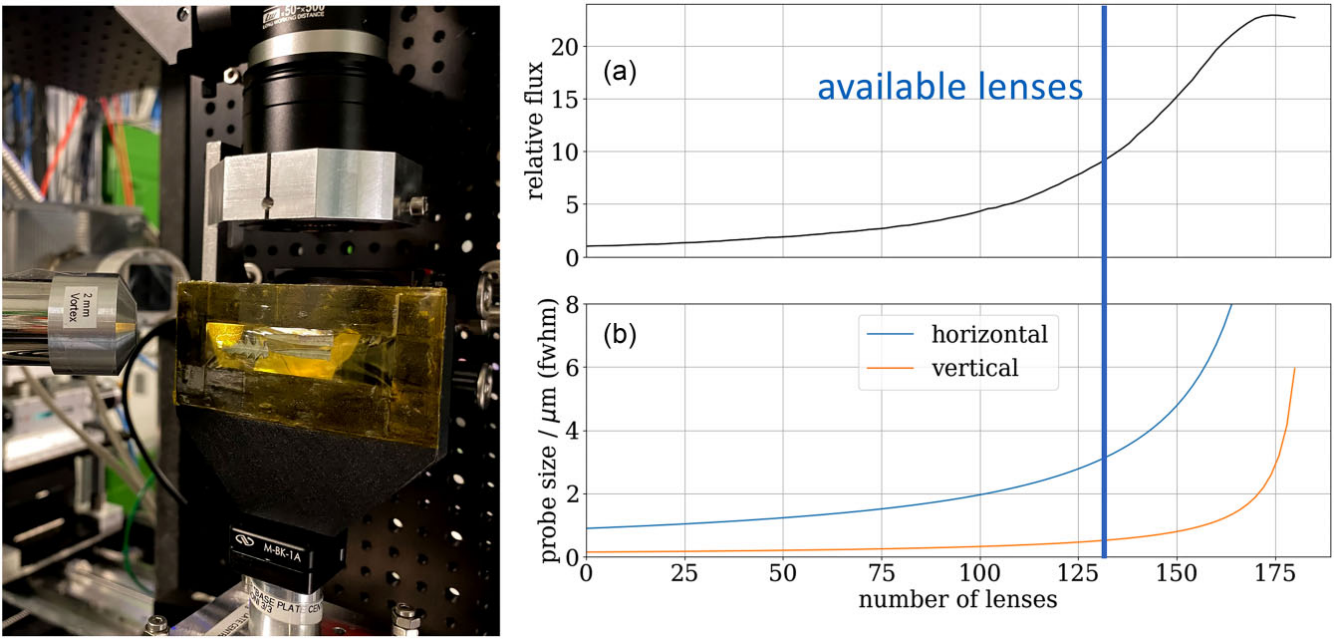
left: Image of the high-energy synchrotron µXRF setup with a leaf sample between Kapton foil. An SDD is placed downstream of the sample at an angle of 315° to the beam and an in-line microscope is placed upstream. Right: Plots of (a) Gain and (b) size of the focused beam as function of the number of applied prefocusing CRL (Be, 1500 µm apex radius of curvature) based on Ray tracing simulations. In the experiment the equivalent of 130 prefocusing CRL were used.

*Dicranopteris linearis* plants were sampled *in situ* on an REE mining site in China. Fresh hydrated specimens and freeze-dried specimens were investigated. The latter were prepared by rapidly pressing fresh plant samples against a solid copper block (∼2 kg) cooled by liquid nitrogen to effect rapid freezing, and then lyophilized in a freeze-dryer (−85°C, <0.003 mbar) for 48 h. The hydrated specimens were cut from living plants immediately before the µXRF analysis and mounted between a sheet of Kapton sticky tape as backing and covered by a sheet of polypropylene thin film (Ultralene, 6 µm thickness) placed over a plastic frame for the analysis. The samples did not dehydrate during the analysis because they were contained in a tight thin film sandwich (see Fig. 1 left).

### Synchrotron µXRF experiment

The X-ray fluorescence microscopy experiments were undertaken at PETRA III (Deutsches Elektronen-Synchrotron DESY), a 6 GeV synchrotron radiation source, specifically at the microprobe end-station, located 93 m from the source, at the Hard X-ray Micro/Nano-Probe beamline P06.^21^ P06 is an undulator beamline equipped with a cooled double-crystal monochromator with Si(111) crystals. The microprobe endstation uses Kirkpatrick–Baez (KB) mirrors for focusing up to a maximum energy of 21 keV, limited by the rhodium coating of the mirrors and a fixed incident angle 2.7 mrad of the K/B mirrors.^22^ For higher energies the KB-based optics setup can be exchanged with an optics setup based on CRL.^23^ The end-station focusing optic consists of a stack of 149 Beryllium CRL (RXOPTICS, Germany) with 350 µm diameter circular aperture and 50 µm apex radius of curvature. A hexapod on top of a translation with 1000 mm travel allows precise and stable alignment of the lens container to the beam. The Si(111) monochromator provides a stable beam with homogeneous wavefront across the aperture of the CRL. The lenses were able to focus the beam at 44 keV at 994 mm focal distance down to 2.7 × 1.2 µm at a flux of 1.5 × 10^9^ ph s^−1^ as determined with an Au wire cross knife-edge scan and a calibrated transmission diode. We were limited to 44 keV excitation (just reaching the absorption edge of Nd at 43.57 keV) due to the use of Si(111) monochromator, the number of available CRLs and the maximum distance of the CRLs to the sample. Another beamline optical component is a pre-focusing transfocator located 43 m from the source. The transfocator contains multiple sets of CRLs with the focusing power up to the equivalent of 130 Be lenses with apex radii of 1500 µm that can be inserted independently. The transfocator gives P06 the option to increase the intensity of the probe at the expense of increased probe size. The pre-focusing increased the photon flux to 5.4 × 10^9^ ph s^−1^, a factor of 4 compared to the non pre-focused beam and increased the probe size to 6 × 2.4 µm. This condition was used for the shown measurements at high excitation energy. Ray tracing simulations (shadow) showed that at 44 keV, the highest flux is achieved by inserting all 130 available pre-focusing CRLs. In principle, the flux could be increased even further with more lenses when they are available (see Fig. 1 right). XRF detection was performed using a Vortex-EM SDD with 2-mm-thick chip (Hitachi High-Tech, USA) in combination with an Xspress 3 pulse processor (Quantum Detectors, UK). The XRF detector was placed in an 315° geometry, with the forward direction of the X-ray beam defined as 0°, looking upstream onto the sample backside, which is itself facing the beam (see Fig 1 left). The increased thickness of the Si sensor material compared with a standard SDD enhances the sensitivity of the detector for 37 keV radiation (Nd-Kα) from 6.5% (0.35 mm thickness) or 9.3% (0.5 mm thickness), respectively, to 32.5% absorption at 2 mm thickness. SDD technology for sensor elements with higher absorption, such as Ge or CdTe, would be useful but is not available yet.

The high excitation energy experiments were complemented by similar measurements at 16 keV on the same sample using the standard setup with KB mirror focusing (2.1 × 3.6 µm focus size, 1.25 × 10^11^ ph s^−1^, with pre-focusing). At low energy excitation two multi-element XRF detectors were used in parallel: a 4-element SDD detector (350-µm-thick chip with 170 mm^2^ combined active area for four elements, Hitachi Vortex ME4) in 45° geometry and a prototype 16-element SDD Ardesia detector (800-µm-thick chip with 324 mm^2^ combined active area for all 16 elements, Politecnico Milano, Italy^24^ in 315° geometry with Xspress 3 pulse processors. The detector assembly allows large solid angle detection and processing of several million counts/s. All measurements were done in continuous scanning mode with dwell times between 4 and 200 ms. In this study interval and step sizes of 15–20 µm where applied which are larger than the beam size, but not limiting the spatial resolution. We investigate thick leaf samples in 2D projection. Due to the penetrative nature of hard X-ray radiation, information from different depth and cells of different plant organs overlap. Therefore, the sample limits the resolution in the element maps and smaller step sizes than 20 µm do not provide higher resolution in the measurement.

### Data processing and statistical analyses

The XRF data was processed using nonlinear least-squares fitting as implemented in PyMCA.^25^ Elemental calibration was performed using calibration foils (ZnTe and CsBr, Micromatter Technologies Inc., Canada) and fundamental parameters from xraylib.^26^ This produced 32-bit .tiff-images with pixel values corresponding to micrograms per square centimeter areal density of the element in question. The figures were prepared in ImageJ^27^ by changing the lookup table (LUT) to either grayscale or “Fire,” adjusting of the maximum values and adding length scales. Self-absorption correction was not necessary for REE K-lines. For REE L-lines no self-absorption correction was applied due to the undefined geometry of the sample shape.

## Results and discussion

### Distribution of LREEs in the *D. Linearis* specimens

**Fig. 2.**
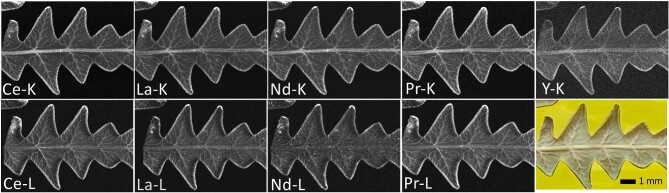
Synchrotron µXRF maps of Ce, La, Nd, and Pr in freeze-dried *Dicranopteris linearis* pinnules at 16 [bottom row (except photo), all L-lines] and 44 (top row, all K-lines) keV incidence energy. Scan resolution of 11 and 16 µm, a dwell time of 4 and 150 ms, respectively. Corresponding concentrations are reported in Table 2 (scan# 42 for 16 keV and scan #48 for 44 keV).

A freeze-dried *D. linearis* pinnule sample was measured with synchrotron µXRF imaging at 16 and 44 keV incidence energy for comparison of the quality of semiquantitative imaging of the LREEs, Ce, La, Nd, and Pr via L-shell and K-shell excitation. The LREE maps recorded under both conditions are shown in Fig 2. Y could only be excited at the high-energy condition. The mean elemental composition of the *D. linearis* plant material and the soil (mg kg^−1^) was determined by ICP–OES and ICP–MS and shown in Table 1. The total concentration of REEs in the pinnules was 1960 ± 102 mg kg^−1^ of which La was the most abundant REE (579 ± 86.9 mg kg^−1^) followed by Nd (532 ± 23.5 mg kg^−1^) and Ce (201 ± 4.71 mg kg^−1^). We also quantified the bulk concentrations in using µXRF imaging by averaging the prevailing concentrations over a large area of the respective sample, shown in Table 2. The values obtained from the 16 keV XRF measurements are consistently lower than those for 44 keV, which is likely caused by self-absorption, which is less pronounced in the high energy measurements. The ICP–OES/MS and XRF samples are not identical, which explains part of the differences in the values obtained. The samples are also highly variable, as can be seen from comparing scans #37 and #48, in which the Ce concentrations differ by approx. two-fold (Table 2).

**Table 1.** Elemental composition of the *Dicranopteris linearis* plant material and the soil (mg kg^−1^) as determined by ICP–OES and ICP–MS

	Total REEs	La	Ce	Nd	Y	Ca	Fe	Mn	Zn
Soil	562 ± 70.4	137 ± 17.0	193 ± 26.0	108 ± 13.4	94.3 ± 12.2	163 ± 0.59	26400 ± 98.1	403 ± 3.10	102 ± 2.66
Root	797 ± 61.4	226 ± 10.4	80.5 ± 29.6	214 ± 11.0	85.1 ± 16.8	761 ± 6.17	589 ± 4.61	543 ± 7.87	27.4 ± 0.39
Pinnule	1960 ± 102	579 ± 86.9	201 ± 4.71	532 ± 23.5	191 ± 74.5	1020 ± 137	185 ± 108	891 ± 110	33.5 ± 0.52

**Table 2.** Calculated bulk concentrations (mg kg^−1^) as calculated from the µXRF maps for selected relevant elements with average noise subtracted (0.03 cm average specimen thickness, 0.8 g cm^2^ specimen areal density)

Energy									
(keV)	#Scan	La	Ce	Nd	Y	Ca	Fe	Mn	Zn
16	42	125.7	100.7	96.4		391.4	47.04	531.7	18.3
44	37	182.3	112.2	273.3	9				
44	48	171.3	220.6	189.1	16.3				

The distributions of Ce, La, Nd, and Pr are very similar between elements and also for both excitation conditions. For high energy excitation, Y could also be detected, and the Ba distribution is shown for comparison. Low energy excitation provides access to additional lighter elements (K, Ca, Cr, Mn, Fe, Cu, Zn, Pb, Rb), which are shown in Supplementary Fig. S1. All these elements are enriched in minor/secondary veins, necrotic areas, and in the margins (tips) of the pinnules. This observation confirms earlier studies of Liu *et al*. who reported that La and Ce were detected in the conducting tissues, epidermis and in necroses in *D. linearis*.^19^ It can be observed that the contrast between the enriched parts and the surrounding tissue of the pinnule is element specific and varies also between the LREEs. The plants seem to have evolved the ability to differentiate between LREEs at least quantitatively.

**Fig. 3.**
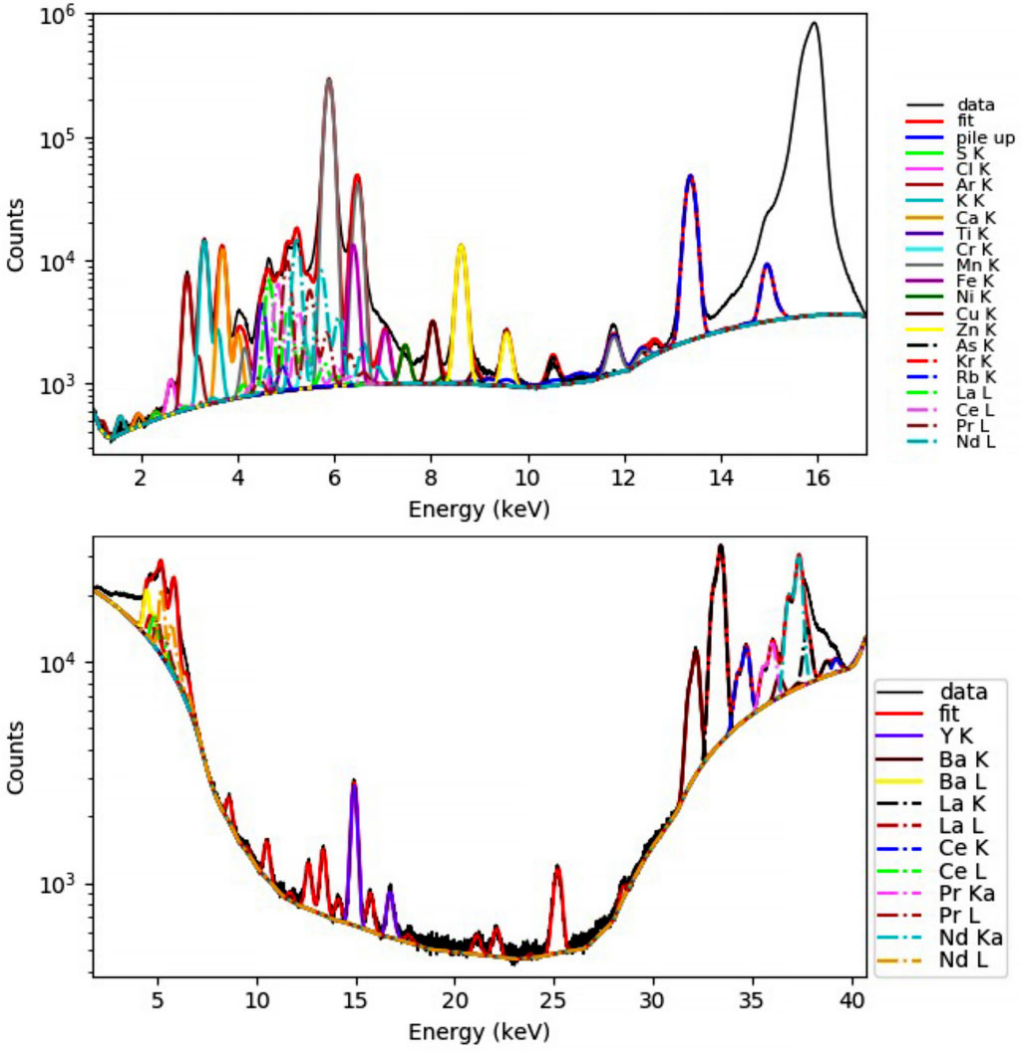
Representative XRF sum spectra of XRF scans on *Dicranopteris linearis* samples. Top panel spectrum was acquired at 16 keV and bottom panel spectrum was acquired at 44 keV. Note the interference of REE L lines (dotted) with transition metal K lines (top spectrum). The REE K lines are well separated (bottom spectrum).

The ratio of REE areal densities is similar for L-line and K-line for Ce, La, and Pd, but different for Nd. This is probably due to nonaccurate fitting of the Nd-L lines due to overlap with transition metal K-lines (Mn, Fe). Figure 3 shows sum spectra of the same necrotic area from the measurement at 16 and 44 keV. Where the LREE K-lines are well separated and can be peak fitted accurately, the LREE L-lines overlap with transition element K-lines and fitting is less clear. Part of the Nd-L line intensity may have been assigned to Cr and Mn K-lines. Another advantage of LREE K-line analysis is that due to their high energy, no absorption correction of the fluorescence signal is required even for millimeter thick tissues. In contrast, the LREE L-lines already suffer from extinction within a few hundreds of micrometers for hydrated samples and into the millimeter range for dried samples. The information depth is different, which explains the higher intensity of surface defects for LREE L-line analysis.

**Fig. 4.**
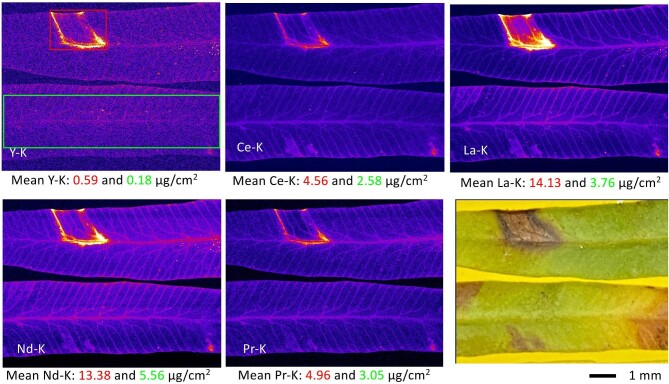
Synchrotron µXRF maps of Y, Ce, La, Nd, and Pr of fresh/hydrated *Dicranopteris linearis* pinnules at 44 keV incidence energy, scan resolution of 20 µm and dwell time of 200 ms. The red and green boxes indicate the area of necrotic and regular tissue for which mean element areal densities were calculated.

The experiment shown in Fig 4 demonstrates that K-shell LREE excitation XRF works as well also for fresh, i.e. hydrated *D. linearis* pinnule samples. The element specific contrast variation between regular tissue and necrotic areas is again obvious. Whereas the concentration ratio between necrotic areas and regular areas is about 3:2 for Ce, Nd, Pr, Y (and also Ba), it is almost 3:1 for La. A significant difference between the measurements at 44 and 16 keV is the dwell time required for a given contrast of the LREEs. Whereas for the measurements at 16 keV a dwell time of 4 ms was sufficient, for 44 keV excitation dwell times of 150–200 ms were appropriate. The increased dwell time is due to the lower photon flux in the focused beam (1/20) on the one hand and the X-ray detectors [single element SDD vs. (16 + 4) elements SDD (reduced solid angle (1/3), reduced sensitivity at 30–35 keV (1/3)] on the other hand. At REE L-line energies, all XRF photons are absorbed in the detector material and absorption in air or detector window is not significant. Note that, although the absorption cross-section for LREEs is lower at 44 keV compared to 16 keV, the XRF production cross section for LREE K-lines at 44 keV is a few times higher than for LREE L-lines at 16 keV.^26^ This is due to the higher X-ray fluorescence yield for LREE K-shell excitation and therefore not a reason for the increase in dwell time. Neglecting limits of flux, X-ray focusing and detection, high-energy excitation (about 90 keV) would be the ideal condition for imaging trace level heavy metals. Light matrix elements were much less efficiently photo-ionized and electron emission would be minimized resulting in lowest detection limits and smallest radiation damage.

For the given pixel size of the scans of 15–20 microns, radiation damage was shown not to be limiting the experiment. The freeze-dried samples are relatively radiation hard due to the absence of water, even at 500 ms dwell time. Even for fresh hydrated samples the radiation dose of ∼0.1 kGy (estimation based on Nicolas *et al*.^28^) should not cause severe radiation damage (refer to Jones *et al*.^29^ who showed that 4.1 kGy is the critical dose limit above which damage occurs), and there were no discoloration or morphological changes after the scans. Moreover, radiation damage may not affect the distribution of La, Ce, Pr, and Nd in this particular case, because (i) *D. linearis* has very low water content and consists of >30 wt% Si in the form of silicates and (ii) REEs are present in *D. linearis* in the form of water insoluble complexes.^13^ Previous studies found that a large proportion of *D. linearis* leaf REE distribution was restricted within the veins, and the remaining REEs were mostly transported to the leaf epidermis or necrosis (if there were any).^19^ This adsorption ability of cell walls for REEs likely leads to unselective fixation of REEs, and subsequently, similar localizations of REEs on the leaf at the level of tissue.^30^

### Outlook and conclusions

The use of high-energy K-line excitation of REEs under the experimental conditions at P06 described here is relatively slow (per pixel dwell in the order of 100–500 ms) and is appropriate for highly concentrated samples (>1000 mg kg^−1^ tREEs bulk concentration). If interfering elements (Cr–Ni) are not abundant, or at least 10–100-fold lower than the REEs in the sample in question, then L-line excitation of REEs using lower energy (11–16 keV) is much more efficient permitting 5–20 ms dwell times per pixel. However, this may be problematic in many plant samples as co-localization of La-Ce-Sm-Gd occurs in *D. erythrosora*.^18^ Note also that the information depth into the sample (absorption length of characteristic X-ray fluorescence) is different for L-shell and K-shell excitation. The extinction length in hydrated samples is 150 µm for L-lines, but in the centimeter range for K-lines. K-shell excitations allow XRF measurement through the volume of millimeter thick samples and, for example XRF tomography of REEs of frozen hydrated plant samples, as demonstrated recently for the Cd distribution in soybean roots.^31^

## Supplementary Material

mfad050_Supplemental_File

## Data Availability

The data that support this study will be shared upon reasonable request to the corresponding author.
