# Supplementary material for: High-energy interference-free K-lines synchrotron X-ray fluorescence microscopy of rare earth elements in hyperaccumulator plants
Source: Metallomics. 2023 Aug 17;15(9):mfad050. doi: 10.1093/mtomcs/mfad050 (PMC10496025; doi:10.1093/mtomcs/mfad050)
Supplement: mfad050_Supplemental_File [file mfad050_supplemental_file.pdf]

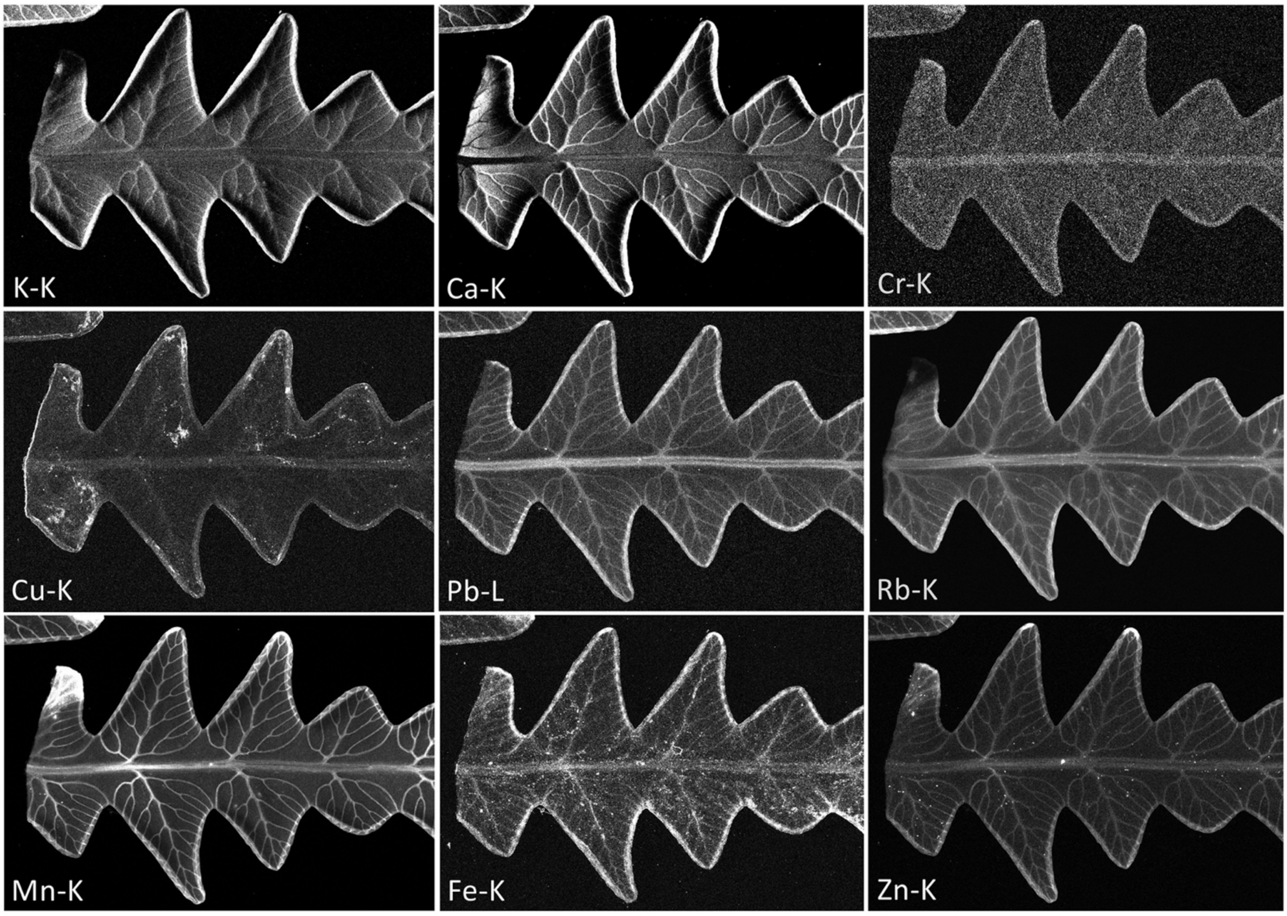

Figure S1. Synchrotron µXRF maps other elements of the same scan as shown in Fig 2 of freeze-dried *Dicranopteris linearis* pinnules at 16 keV incidence energy. REE L-lines beyond Pr-L could not be fitted because of low concentrations and strong interferences with intense Fe, Ni, Cu and Zn K-lines. Refer to Table 2 scan #42 (16 keV) for concentration values.
